# Race and Sex Disparities Among Emergency Medicine Chief Residents

**DOI:** 10.1001/jamanetworkopen.2024.32679

**Published:** 2024-09-24

**Authors:** Jennifer W. Tsai, Mytien Nguyen, Sarah N. Dudgeon, William McDade, Jung G. Kim, Pooja Agrawal, Dowin Boatright

**Affiliations:** 1 Department of Emergency Medicine, Yale University School of Medicine, New Haven, Connecticut; 2 St Joseph’s Medical Center, Stockton, California; 3 Kaiser Permanente, East Bay, California; 4 Department of Immunobiology, Yale University School of Medicine, New Haven, Connecticut; 5 Department of Laboratory Medicine, Yale University School of Medicine, New Haven, Connecticut; 6 Accreditation Council for Graduate Medical Education, Chicago, Illinois; 7 Ronald O. Perelman Department of Emergency Medicine & Institute for Innovations in Medical Education, New York, New York; 8 New York University Langone Health, New York

## Abstract

**Importance:**

Physicians who belong to minoritized racial and ethnic groups remain underrepresented and underpromoted. Serving as a chief resident is an important position of leadership and prestige, and indicates a benchmark for future professional success. However, it is unknown if disparities in race and/or sex exist in the chief resident selection process.

**Objective:**

To describe race, ethnicity, and sex of emergency medicine (EM) chief residents and determine the association of racial identity and the intersectionality of race and sex for selecting chief residents in US emergency medicine departments.

**Design, Setting, and Participants:**

This cohort study analyzed data collected from the Association of American Medical Colleges and the Electronic Residency Application Service in the graduating classes of 2017 and 2018. Data were analyzed between December 2021 and January 2023.

**Main Outcomes and Measures:**

Relative risk (RR) of selection for chief residency for Black, Asian, and Hispanic EM residents in comparison with White counterparts.

**Results:**

Among 3408 studied residents, 738 (21.7%) served as chief resident (2253 male [66.1%]; 451 Asian [13.2%], 144 Black [4.2%], 158 Hispanic [4.6%], 239 more than 1 race [7.0%], 46 other [1.3%], and 2370 White [69.5%]). Of chiefs, 81 (11.0%) identified as Asian, 17 (2.3%) as Black, and 26 (3.5%) Hispanic. Asian residents were 78% (95% CI, 63%-96%) as likely to be promoted to chief resident compared with White peers, and Black residents were 51% (95% CI, 32%-80%) as likely as White residents. In our fully adjusted model, racial differences remained significant for Black residents, who were half as likely as white residents to be selected for chief residency (adjusted risk ratio [aRR], 0.55; 95% CI, 0.36-0.82). Overall, White women were most likely to be selected for chief residency and 20% more likely to be selected than White men counterparts (aRR, 1.20; 95% CI, 1.03-1.39). In comparison, women underrepresented in medicine (a category that included residents identified as Black, Hispanic, American Indian or Alaskan Native, and Native Hawaiian or Other Pacific Islander) were least likely to be selected for chief promotion, and 50% as likely to be selected for chief resident compared with White men (aRR, 0.50; 95% CI, 0.06-0.66).

**Conclusions and Relevance:**

In this 2024 nationally representative study of EM residents, chief promotion was lower among residents identifying as Asian or Black, and in particular, women underrepresented in medicine. This study’s findings suggest further review of chief resident selection process by residency programs and accreditation bodies is needed to ensure workforce equity for promotion and opportunities for leadership.

## Key Points

**Question:**

Do racial disparities for selecting emergency medicine chief residency exist?

**Findings:**

This cohort study of 3408 US emergency medicine residents found significant inequities in chief resident selection across race and sex. Residents who are Black and woman residents from racial and ethnic groups that are underrepresented in medicine (URIM) were less likely to be selected as a chief resident in adjusted analyses, but White women residents were found 1.2-times more likely than White men to be chief resident, and White men were twice as likely to be chief resident compared with URIM women.

**Meaning:**

Selection for chief resident may be vulnerable to biases, which may affect career prospects for physicians and especially women URIM physicians.

## Teaser

This cohort study of US emergency medicine residents graduating in 2017 and 2018 analyzes the selection of chief residents by race, ethnicity, and sex.

## Introduction

Chief residents are recognized as leaders within residency programs, and benefit from the position through financial compensation, departmental influence, career advancement opportunities, and prestige.^1,2,3^ While chief residents are supposedly chosen based on demonstrated leadership, clinical skills, teaching abilities, and managerial acumen, there are no systematically defined criteria or responsibilities for their selection.^2,3,4,5,6^

Despite the profound impact on professional advancement, little is known about differences in chief selection by race or ethnicity. This lack of knowledge exists amidst a backdrop of significant racialized inequities that permeate across the health professions workforce.^7,8,9,10,11,12,13,14,15^ Racialized disparities in clerkship grades, honor society inductions, and letters of evaluation impact medical trainees of color at the undergraduate level,^16,17,18^ while inequities in advancement, National Institutes of Health (NIH) funding, and promotion hinder clinicians even after graduation.^19,20,21,22,23,24,25^ These existing disparities for race-based mistreatment and lower perceived professional performance^19^ affect the physician of color workforce: from greater rates of depression to job burnout.^26,27,28,29^

Examination of racial and/or ethnic disparities in chief resident selection offer an opportunity to determine if similar inequities for professional recognition exist in graduate medical education (GME). Reports have found that women are less likely to be selected for chief residency, and that even when selected, women report faculty are less helpful and supportive to them vs men counterparts.^1,2,30,31^ In addition, while previous studies of chief selection have stratified data by sex, almost none have examined what has been described as the “double jeopardy” of racism plus sexism against women who belong to minoritized racial and ethnic groups.^32^

Because the recruitment and promotion of a diverse physician workforce is a central issue to ensure health equity, investigating gendered and racial disparities within chief resident selection is critical and timely.^33^ Given that inequities in chief selection may systematically limit opportunities for employment and advancement—including fellowship opportunities and promotion in both community and academic settings—such disparities may amplify ongoing issues with workforce equity in ways that extend across generations. This study addresses the paucity of research in this area by examining the association of race, ethnicity, and sex for selecting chief residents among a national cohort of EM residents.

## Methods

### Study Design and Data Source

We conducted a retrospective cohort study of EM residents from the graduating class of 2017 and 2018 using the Association of American Medical College’s (AAMC) data services. This included resident demographic data, including race and ethnicity, sex, age, medical school, and US Medical Licensing Examination (USMLE) Step 2 Clinical Knowledge (CK) scores. Race and ethnicity was gathered mostly by self-report, with some third-party reported, and included 7 categories: “Asian,” “Black or African American,” “Hispanic, Latino, or of Spanish Origin,” “Native Hawaiian or Other Pacific Islander,” “American Indian or Alaskan Native,” “White,” and “other.” For intersectional analysis, race and ethnicity was recategorized into 3 categories: White, Asian, and underrepresented in medicine (URIM). URIM includes residents who identified as Black, Hispanic, American Indian or Alaskan Native, and Native Hawaiian or Other Pacific Islander.^34^

This study followed the Strengthening the Reporting of Observational Studies in Epidemiology (STROBE) reporting guidelines for cohort studies. The study was reviewed and deemed exempt from obtaining informed consent by the Yale University institutional review board because it used only deidentified data.

### Study Population

We studied graduates from all US-accredited EM residency programs in 2017 and 2018. We excluded individuals with incomplete Step 2 CK score data and non-US citizens, because race and ethnicity data for these residents were not provided (951 residents [21.8%]). Individuals with more than 1 racial category were recognized as multiracial, while those with missing race or ethnicity data were categorized as unknown race and excluded from analysis (9 residents [0.3%]).

### Statistical Analysis

We compared differences in demographic data characteristics between residents who were selected for chief resident promotion and those who were not using χ^2^ tests for categorical variables. We then used binomial regression to model the relative risk of sex with race and ethnicity, as well as the intersectional sex–race and ethnicity identity, on the likelihood of selection for chief resident.

We also modeled additional factors to account for nuanced differences in selecting chief residents. Prior studies on chief selection have reported sex disparities, so we adjusted for sex and USMLE Step 2 CK score by quartiles as a proxy for medical knowledge. Because promotion as chief resident is decided within residency programs, we also accounted for clustering within each training program by using generalized estimating equations with compound symmetry correlation to model the covariance structure. Given the a priori theorization regarding the double jeopardy of racism and sexism against women of color, we also conducted an intersectional analysis.^32,35,36^ Relative risk ratios (RRs) and 95% CIs were calculated to measure observed associations. All analyses were performed using Stata (StataCorp LLC).

## Results

A total of 3408 residents were included (2253 male [66.1%]; 451 Asian [13.2%], 144 Black [4.2%], 158 Hispanic [4.6%], 239 as more than 1 race [7.0%], 46 as other [1.3%], and 2370 White [69.5%]); 738 (21.7%) were selected for chief residency (Table 1). Of chiefs, 468 (63.4%) were men compared with 270 (36.6%) women. There were differences in our sample of chief residents by race or ethnic identity as compared with the population of those not selected; 548 selected residents (74.3%) were White, 81 (11.0%) were Asian, 26 (3.5%) were Hispanic, and 17 (2.3%) were Black (*P* = .002) (Table 1). More specifically, our unadjusted model found that White residents were significantly more likely to be selected for chief resident: Asian and Black residents were only 78% (95% CI, 63%-96%) and 51% (95% CI, 32%-80%) as likely to be selected for chief resident compared with their White counterparts (Table 2).

**Table 1.** Characteristics of Study Sample

Characteristic	Chief residents, No. (%) (N = 3408)		*P* value
	No (n = 2670)	Yes (n = 738)	
Sex			
Male	1785 (66.9)	468 (63.4)	.08
Female	885 (33.2)	270 (36.6)	
Race or ethnicity			
Asian	370 (13.9)	81 (11.0)	.002
Black	127 (4.8)	17 (2.3)	
Hispanic	132 (4.9)	26 (3.5)	
>1 Race	187 (7.0)	52 (7.1)	
Other^a^	32 (1.2)	14 (1.9)	
White	1822 (68.2)	548 (74.3)	
USMLE Step 2 CK			
176-231	732 (27.4)	148 (20.1)	<.001
>231-242	658 (24.6)	178 (24.1)	
>242-253	682 (25.5)	210 (28.5)	
>253-282	598 (22.4)	202 (27.4)	
Intersectional identity			
White man	1249 (50.9)	346 (51.4)	<.001
Asian man	256 (10.4)	51 (7.6)	
URIM man	148 (6.0)	31 (4.6)	
White woman	573 (23.3)	202 (30.0)	
Asian woman	114 (4.6)	30 (4.5)	
URIM woman	115 (4.7)	13 (1.9)	

Abbreviations: URIM, underrepresented in medicine (Black, Hispanic, American Indian or Alaskan Native, and Native Hawaiian or Other Pacific Islander); USMLE Step 2 CK, US Medical Licensing Examination Step 2 Clinical Knowledge.

^a^ Other includes American Indian and Alaska Native and all other races not otherwise listed.

**Table 2.** Unadjusted and Fully Adjusted Risk Ratio Analysis Relative Risk (RR) for Chief Residency Selection by Demographic Identity

Characteristic	Unadjusted RR (95% CI)	Adjusted RR (95% CI)^a^
Sex		
Male	1 [Reference]	1 [Reference]
Female	1.13 (0.99-1.28)	1.14 (1.01-1.30)
Race or ethnicity		
White	1 [Reference]	1 [Reference]
Asian	0.78 (0.63-0.96)	0.81 (0.65-1.00)
Black	0.51 (0.32-0.80)	0.55 (0.36-0.82)
Hispanic	0.71 (0.50-1.02)	0.76 (0.54-1.07)
>1 Race	0.94 (0.73-1.21)	0.95 (0.74-1.22)
Other^b^	1.32 (0.85-2.05)	1.40 (0.93-2.13)

^a^ Fully adjusted for race and ethnicity, sex, US Medical Licensing Examination Step 2 Clinical Knowledge score, and program clustering.

^b^ Other includes American Indian and Alaska Native, Native Hawaiian and Other Pacific Islander, and other races not otherwise listed.

Our fully adjusted model—which modeled for race and ethnicity, sex, USMLE Step 2 scores, and residency program—demonstrated a significant sex disparity for chief selection that favored women (adjusted relative risk [aRR], 1.14; 95% CI, 1.01-1.30) (Table 2). Racial and ethnic disparities for Black residents remained significant (aRR, 0.55; 95% CI, 0.36-0.82), although significant results were not observed for Asian residents (aRR, 0.81; 95% CI, 0.65-1.00) and remained nonsignificant for Hispanic residents (aRR, 0.76; 95% CI, 0.54-1.07) (Table 2).

Analysis for intersectional identities of both sex and race were found that White women residents compared with White men were considerably more likely to be selected for chief resident (aRR, 1.20; 95% CI, 1.03-1.39) after adjusting for USMLE Step 2 score and clustering of EM program (Table 3). In comparison, URIM women had the lowest likelihood of being selected for chief resident out of any group (aRR, 0.50; 95% CI, 0.06-0.66; *P* = .01). Asian male residents (aRR, 0.80; 95% CI, 0.61-1.05), URIM male residents (aRR, 0.88; 95% CI, 0.63-1.22), and Asian female residents (aRR, 0.99; 95% CI, 0.70-1.38) were not found to be statistically significant.

**Table 3.** Intersectional Analysis for Chief Residency Selection by Intersectional Demographic Identity

Characteristic	aRR (95% CI)
Intersectional identity	
White man	1 [Reference]
Asian man	0.80 (0.61-1.05)
URIM man	0.88 (0.63-1.22)
White woman	1.20 (1.03-1.39)
Asian woman	0.99 (0.70-1.38)
URIM woman	0.50 (0.31-0.82)
USMLE Step 2 CK	
176-231	1 [Reference]
>231-242	1.22 (0.97-1.52)
>242-253	1.32 (1.08-1.63)
>253-282	1.43 (1.16-1.76)

Abbreviations: aRR, adjusted relative risk; URIM, underrepresented in medicine (Black, Hispanic, American Indian or Alaskan Native, and Native Hawaiian or Other Pacific Islander); USMLE Step 2 CK, US Medical Licensing Examination Step 2 Clinical Knowledge.

## Discussion

In this nationally representative study of EM residents, we found significant racial inequities in chief residency selection. In our fully adjusted model and intersectional analysis, significant chief disparities persisted for Black residents and URIM women, respectively. Both groups were half as likely to receive promotion in comparison with White residents and White men, respectively.

We also found significant sex disparities when intersectional identities were analyzed. Of these groups, White women were the most likely to be selected for chief—at rates 1.2-times greater than White male peers—while URIM women were the least likely to be selected, by a ratio of 50%. These findings confirm the importance of recognizing the double jeopardy of intersectional identities; the ways women who belong to minoritized racial and ethnic groups receive compounded, unique insults from axes of race as well as gender.^32,36^ Their marginalization is not merely equivalent to the added effects of racism and sexism, but complicated and deepened by these intersecting identities, which cause unique problems not faced by White women or men of color.^19,21,35^

Our analyses further the discussion about the sources of racial inequities in medical education and whether they are associated with similar mechanisms of interpersonal biases and structural racism that operate implicitly and explicitly in high-stakes decision-making and threaten a sense of belonging within training programs.^37^ Prior research has reported that trainees of color are almost 30% more likely to withdraw from residency, and 8 times more likely to take a leave of absence for performance difficulties.^38^ These factors continue today among underrepresented and underpromoted medical professionals, encompassing notable racial disparities in trainee performance assessments and promotions into other roles, barriers to professional development that parallel the chief resident selection process.^17,18,19,38,39,40,41,42,43,44,45,46^ These biases—and their avoidable, deleterious outcomes—build upon and inform each other. Thus, disparities at each stage of the medical education continuum have the propensity to unfurl and deepen across the trajectory of the medical profession.^41,42,43,44,47,48,49^ Because chief selection is ostensibly informed, in part, by the assessment of clinical skills and abilities to serve as faculty, racial biases that affect performance evaluations—which has already been demonstrated among EM residents regarding Milestone ratings^19^—could plausibly impact chief residency selection.

It is also intuitive that racism at the institutional level informs race-based chief disparities given enduring and extensive racial disparities across economic, educational, and geographic considerations. Greater social and financial stressors may preclude trainees of color from ultimately seeking lower-paying academic jobs. Trainees who belong to minoritized racial and ethnic groups may also know about empirical racial disparities in academic funding, mentorship, and advancement,^22,23,24,25^ and thus have less incentive to vie for a promotion that primarily impacts academic placement. Such considerations underline the importance of analyzing workforce diversity and representation in structural contexts, and demonstrate that disparities in EM chief residency selection must be considered alongside larger aspects of political economy that impact the career choices and professional realities of medical practice.

While prior efforts have focused on bolstering the pipeline of talented clinicians from racial and sexual minority groups entering medicine,^53,54,55^ there remains a need to identify and address structural barriers within medical education that hinder equitable advancement and health equity.^33,56,57,58,59,60^ Biases in promotion at the GME level should be proactively monitored, especially because the manifestation of these multiple inequities can unfairly impact career trajectories in the long term due to its association with networking opportunities, prestige, and leadership development. Beyond ensuring such disparities do not remain unrecognized, further research into causal explanations may also inform policies and interventions that should be implemented to address inequities in resident advancement. For example, our study’s findings underscore the importance of disaggregation, as it demonstrates that White women are actually the most likely intersectional group to be selected for chief residency, even when compared with White men. This juxtaposes concerning disparities for URIM women and emphasizes the importance of intersectional research and its ability to inform targeted interventions.

### Limitations

This study has the following limitations. First, we examined disparities among major racial and ethnic categories as reported by the AAMC, but did not stratify the sample to include more granular racial and ethnic groups due to limitations in study power. This may be important given that data aggregation can obscure more specific racial and ethnic group disparities. For example, it is known that Asian Americans possess high rates of in-group inequities.^50^ Second, we were unable to identify trans and nonbinary residents, who also represent a minoritized group that faces discrimination during physician training and promotion.^51^ Third, our analyses could not account for individuals who might have been offered a chief resident position but declined to accept. Fourth, we attempted in serial modeling to adjust for covariates that may affect chief selection, but other factors—like performance evaluations, In-Training Exam scores, and degree of prior leadership experience—were not available in the dataset. We considered USMLE Step 2 score, which we rationalized could represent test-taking aptitude and knowledge content, although recognize it is not a perfect marker for fund of knowledge, and that years pass between USMLE Step 2 and chief resident selection. Additionally, previous research has demonstrated racial and ethnic differences in USMLE testing,^52^ so adjusting for these scores biases our results toward the null hypothesis. Fifth, statistically significant differences for chief resident selection were not observed for Asian and Hispanic residents compared with their White peers in our fully adjusted model. Our confidence intervals were observed to be wide, with potentially a type II error resulting from a lack of statistical power. This is a widely known issue within disparity studies: greater granularity of race, ethnicity, and gender variables is necessary for more accurate analysis, but stymied by foundational lack of diversity in the health care workforce. Our findings draw from 2017 to 2018 because that was the most complete dataset available to the investigative team. To our knowledge, this is the first reporting on EM chief resident disparities by race and ethnicity; furthermore, a search reveals no comprehensive interventions to correct to address racial disparities in EM chief selection on a national level. Nevertheless, it is possible that the racial and ethnic representation of chief residents may have changed since 2018. Additionally, GME Track’s dataset typically has a response rate of 95% in recent years, and data may be missing for nonresponding programs. Lastly, this study sampled a single medical specialty, which limits findings to emergency medicine, but remains, to our knowledge, the largest study to examine racial and gender disparities in chief resident selection in GME.

## Conclusions

In this nationally representative study of EM residents, we found that relative ratio of chief promotion is meaningfully reduced for residents who identify as Asian or Black, and for URIM women in particular. This study’s findings suggest that individual and systematic internal review of chief resident promotion by residency programs and the ACGME could benefit workforce equity.

## Notes

Supplement.
Data Sharing Statement
