# Error in Author Affiliations

**DOI:** 10.1001/jamanetworkopen.2024.47077

**Published:** 2024-10-31

In the Original Investigation titled “Race and Sex Disparities Among Emergency Medicine Chief Residents,”^1^ published September 24, 2024, there were errors in the affiliations affecting several authors. Dr Tsai’s affiliation with Kaiser Permanente was added, and the location of the Ronald O. Perelman Department of Emergency Medicine & Institute for Innovations in Medical Education is now listed in New York City. This article has been corrected.^1^
