# Supplementary material for: Race and Sex Disparities Among Emergency Medicine Chief Residents
Source: JAMA Netw Open. 2024 Sep 24;7(9):e2432679. doi: 10.1001/jamanetworkopen.2024.32679 (PMC11423172; doi:10.1001/jamanetworkopen.2024.32679)
Supplement: Supplement. — Data Sharing Statement [file jamanetwopen-e2432679-s001.pdf]

Data Sharing Statement

Tsai. Race and Sex Disparities Among Emergency Medicine Chief Residents. *JAMA Netw Open*. Published September 13, 2024. doi:10.1001/jamanetworkopen.2024.32679

Data
Data available: No
